# Supplementary material for: Evaluation of Anticancer Activity of Zhubech, a New 5-FU Analog Liposomal Formulation, against Pancreatic Cancer
Source: Int J Mol Sci. 2023 Feb 21;24(5):4288. doi: 10.3390/ijms24054288 (PMC10002367; doi:10.3390/ijms24054288)
Supplement: Supplementary file 1 [file ijms-24-04288-s001.zip › Supplemental figures.pdf]

Supplementary Figures

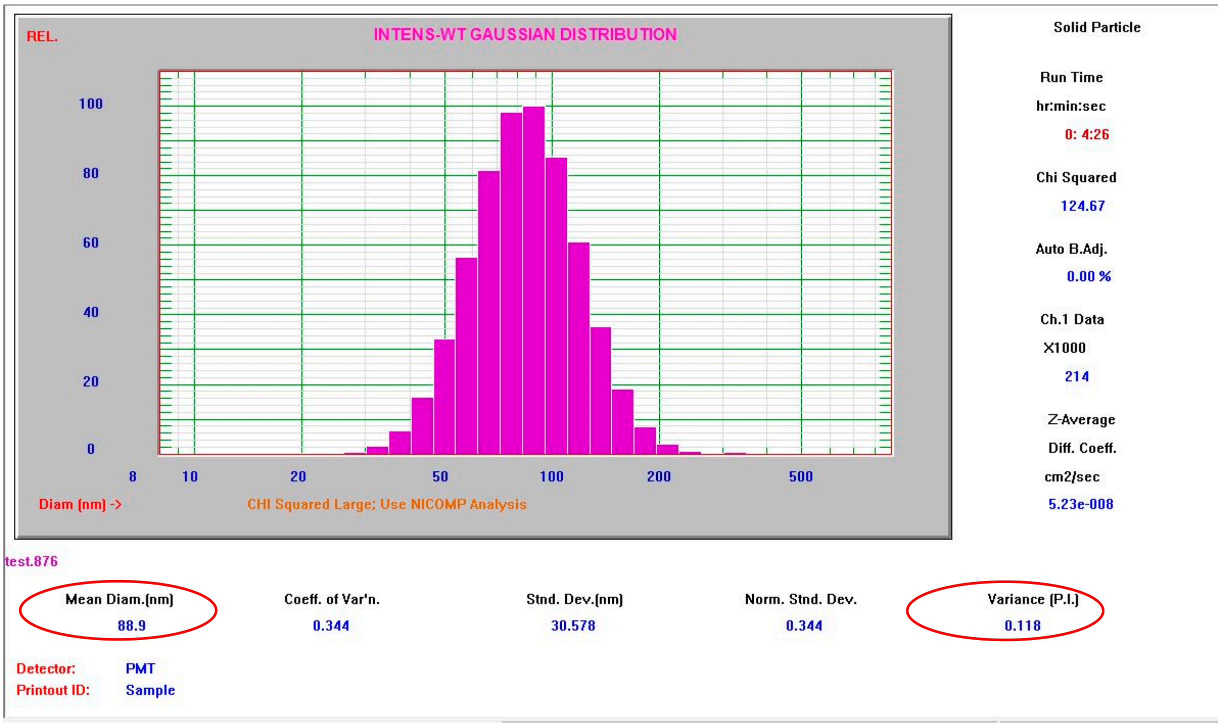

Figure S1: Blank Liposomal formulation (LnP) particle size and PDI (Variance) distributions

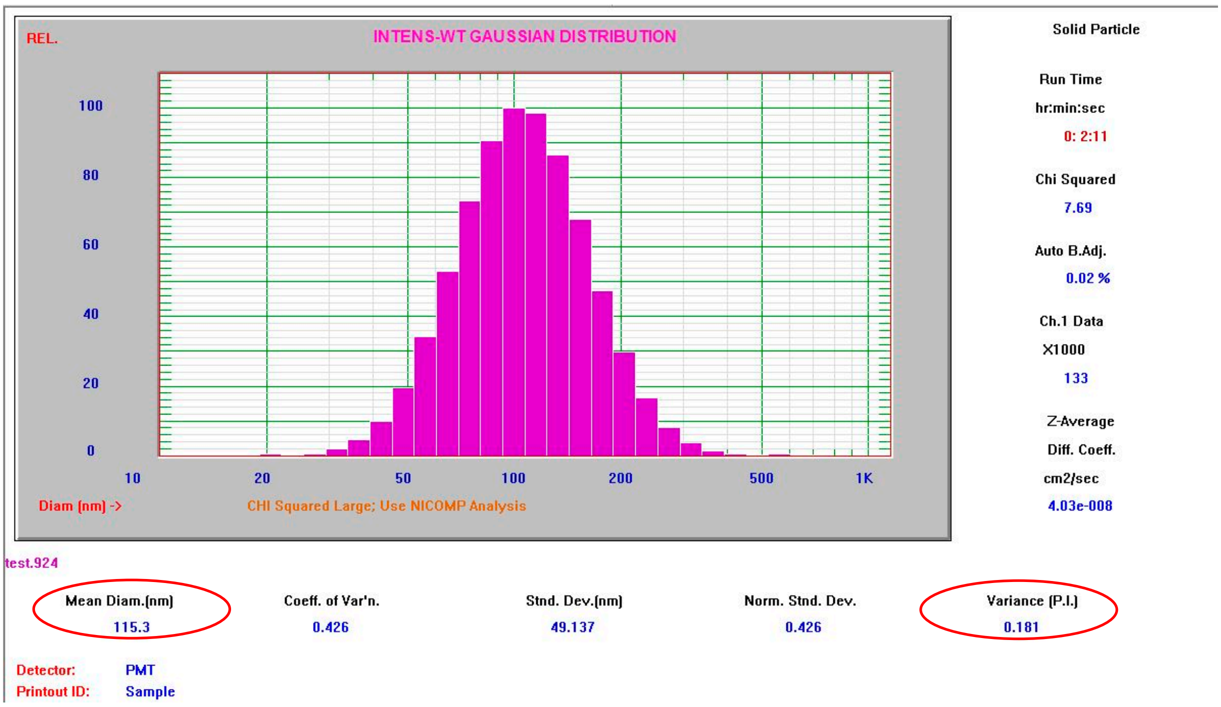

Figure S2: MFU-loaded Liposomal formulation (Zhubech) particle size and PDI distributions

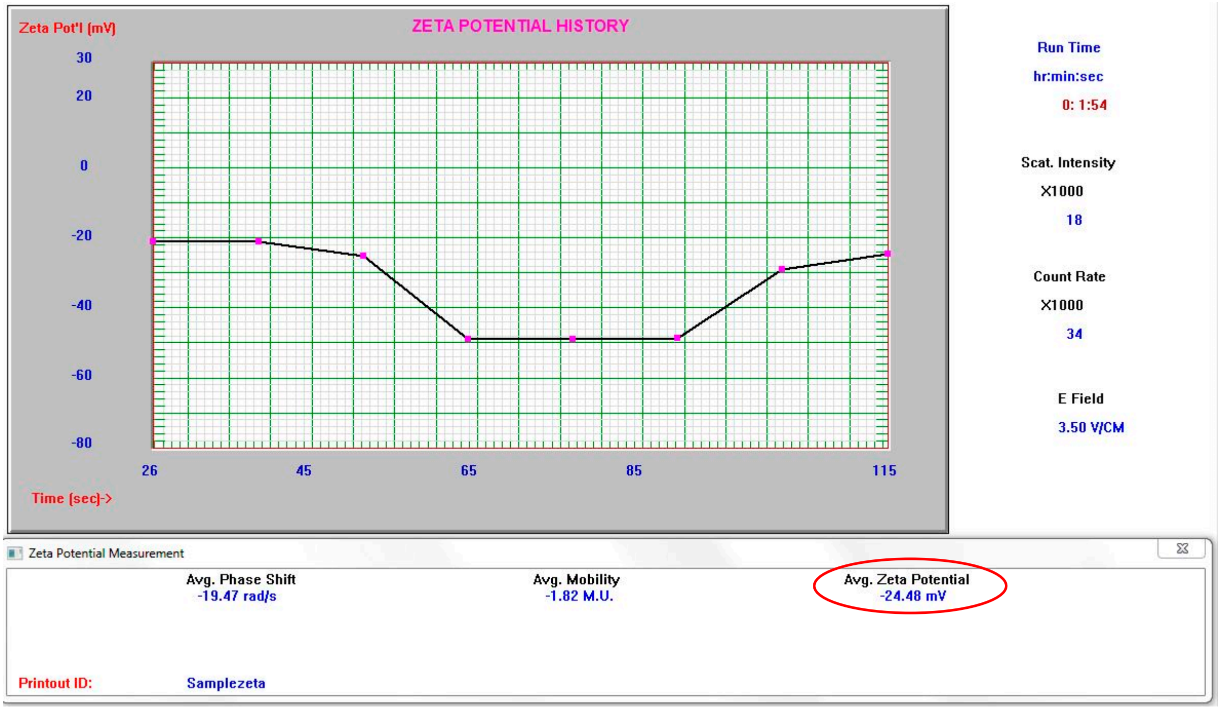

Figure S3: Zeta Potential of MFU-loaded (Zhubech) liposomal formulation]

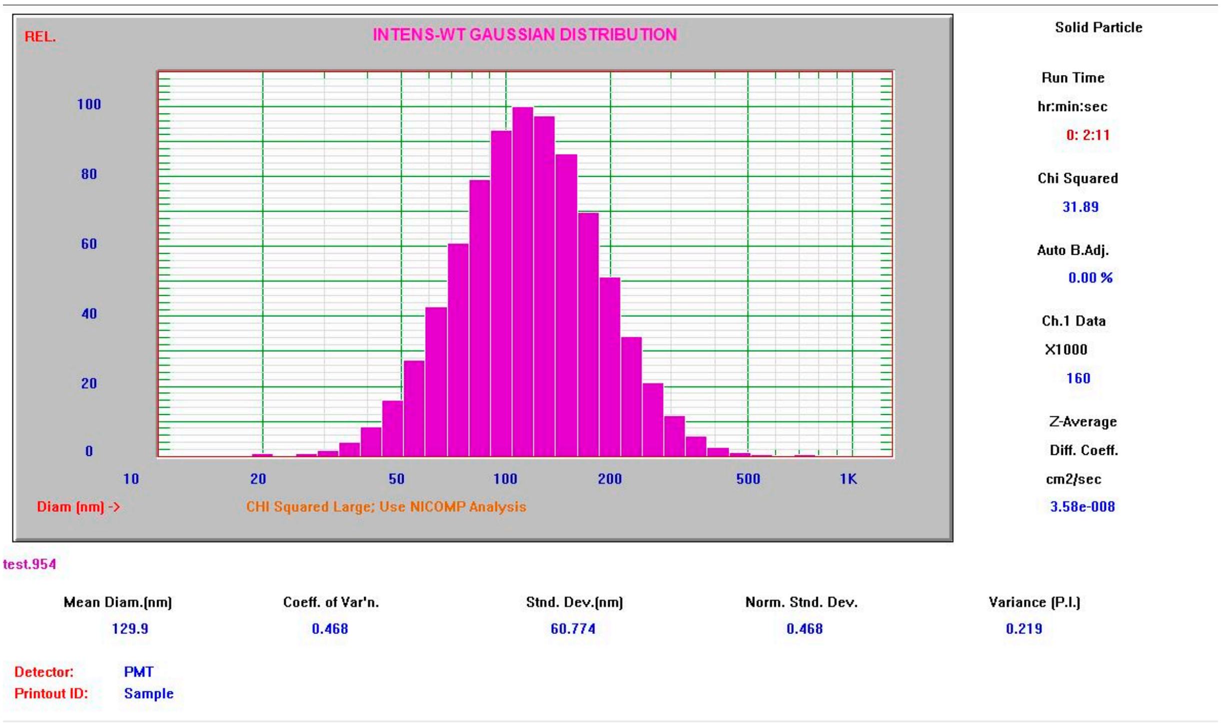

Figure S4: Particle size distribution and PDI of Zhubech at day 30 and at room temperature (25˚C)

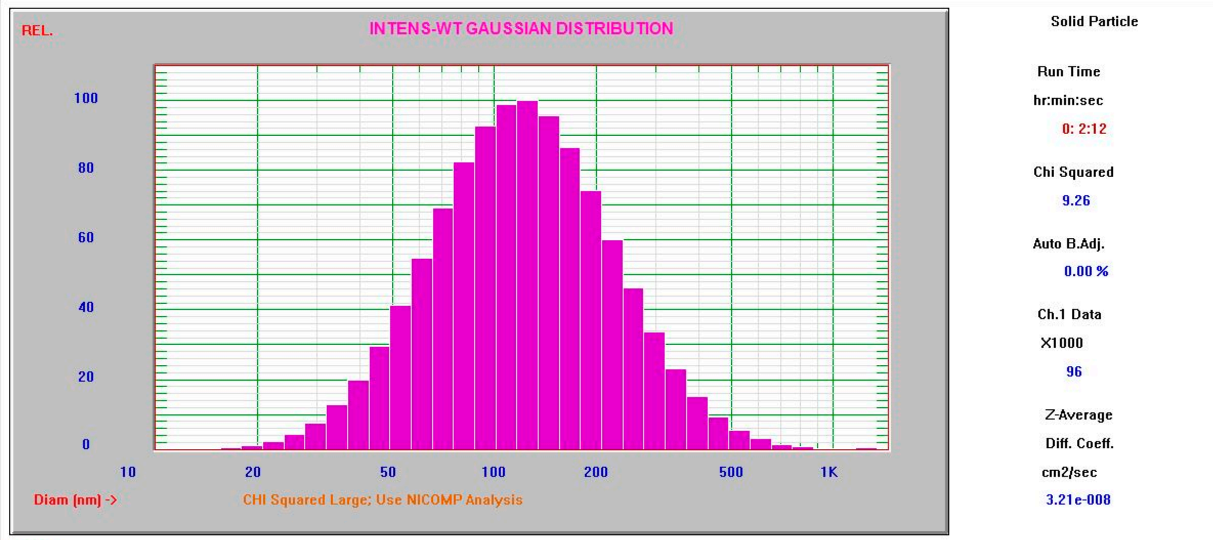

test.948

Mean Diam.(nm)	Coeff. of Var'n.	Stnd. Dev.(nm)	Norm. Stnd. Dev.	Variance (P.I.)
145.0	0.615	89.169	0.615	0.378

Detector: PMT
Printout ID: Sample

Figure S5: Particle size distribution and PDI of Zhubech at day 60 at room temperature (25˚C)

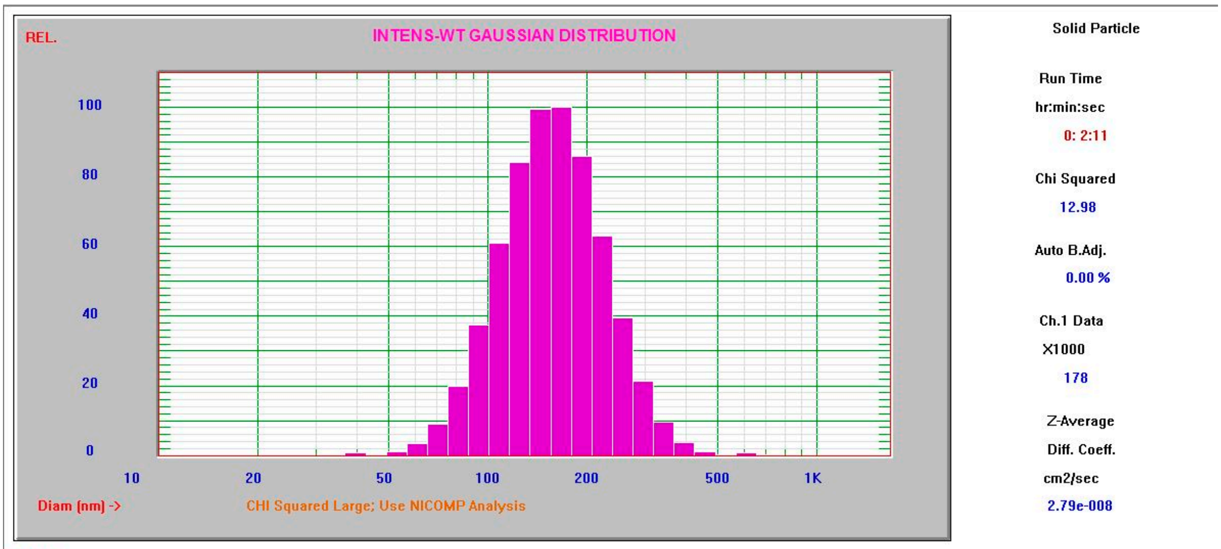

test.990

Mean Diam.(nm)	Coeff. of Var'n.	Stnd. Dev.(nm)	Norm. Stnd. Dev.	Variance (P.I.)
166.7	0.362	60.347	0.362	0.131

Detector: PMT
Printout ID: Sample

Figure S6: Particle size distribution and PDI of Zhubech at day 90 and at room temperature (25˚C)

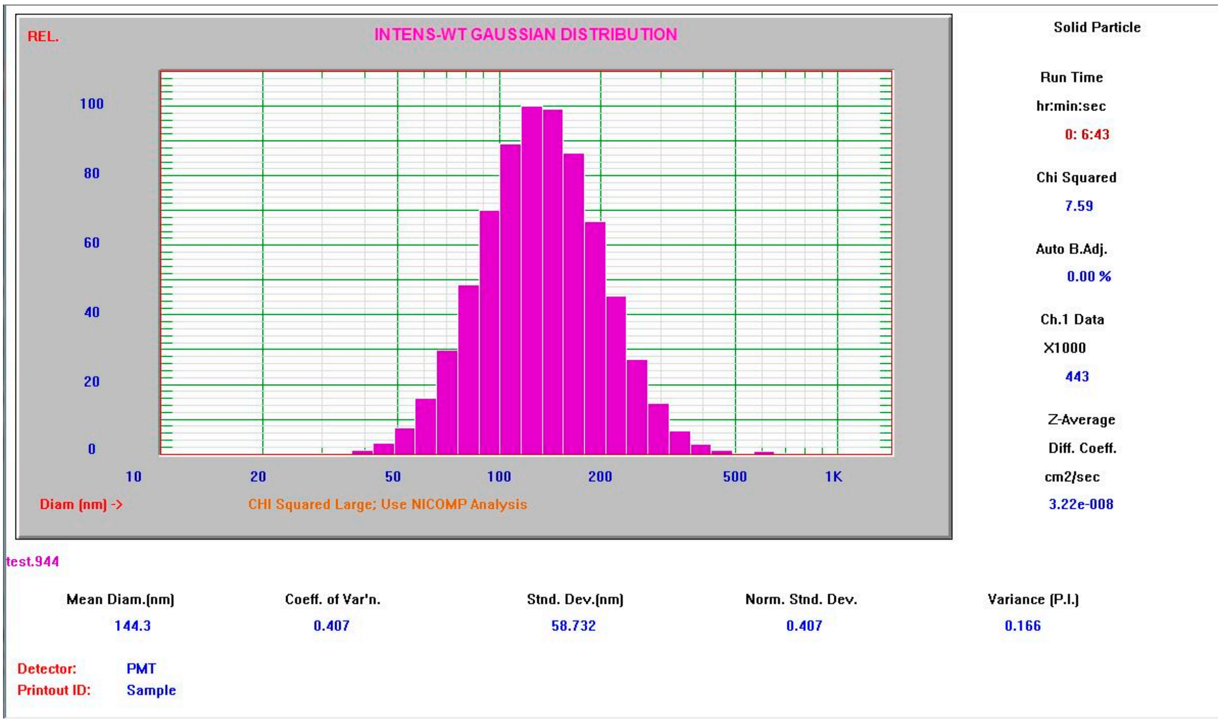

Figure S7: Particle size distribution and PDI of Gdhx-loaded liposomal formulation (Gdhex-LnP)

Supplemental Video S1

Panc-1 cancer cells were grown in 6-well plates (with coverslips) at a cell density of 2×10^3 for 24 h at 37 °C. The cells were then treated with Rhodamine-labeled LnP in growth media. After 12 and 24 h, Rhodmaine-LnP was removed, and the cells were gently washed twice with PBS. Next, 5 µg/ml of DAPI dye was added for nuclear staining; the cells were fixed using 4% paraformaldehyde, then mounted and imaged using Leica SP2 Multiphoton system (confocal microscope). The video taken further demonstrates the orientation of the dye-loaded nanoparticles in a 3D style showing the colocalization of the rhodamine-liposomal formulation in the cytoplasm. The accumulation of labeled liposomes in perinuclear vesicles suggests the accumulation of formulation in the endosomal/lysosomal compartment in a time-dependent manner.
